# Are artificial intelligence chatbots safe for suicide risk assessment? A narratively synthesized review of current evidence

**DOI:** 10.1017/gmh.2026.10260

**Published:** 2026-06-25

**Authors:** Khaled Elbarbary, Amani Alfudoul, Ahmed Amir Samir, Husam Aldean H. Hussain, Balgees Altayib, Amjed Marzouq Abdelqader Abdel Al, George Jabrieh, Rahaf Shaban, Sheikh Shoib, Abdel-Hady El-Gilany

**Affiliations:** 1 Mansoura University Faculty of Medicine, Egypt; 2 Medical Biotechnology and Translational Medicine, University of Milan, Milan, Jordan; 3 Al-Azhar University Faculty of Medicine, Egypt; 4 Jordan University of Science and Technology, Jordan; 5 University of Bahri, Sudan; 6 Palestine Polytechnic University, Palestinian Territory, Occupied; 7 Yeni Yuzyil University, Türkiye; 8 Department of Health Services, Srinagar, Kashmir, India

**Keywords:** suicidal ideation, chatbots, artificial intelligence, natural language processing, mental health services

## Abstract

Suicide is a significant global public health concern, and conventional clinical risk assessment is constrained by workforce availability, scalability, and clinician variability. The classic suicidality risk evaluation is largely dependent on clinical judgment, which, although helpful, can demonstrate a ceiling effect in its predictive validity. AI-driven chatbots, conversational systems that engage users in real-time natural language dialog, have emerged as candidate tools for augmenting suicide risk detection and prevention. This narrative review aimed to: evaluate the performance of AI-driven chatbots in detecting and assessing suicidal ideation relative to clinical benchmarks; examine the effectiveness of chatbot-based interventions for suicide prevention; and identify ethical, cultural, and implementation challenges limiting clinical translation. Six electronic databases were searched, with the initial search conducted in 2024 and updated in 2026 through targeted monitoring, with no upper cutoff date applied. A thematic narrative synthesis approach was applied across five domains. Eleven primary studies met eligibility criteria and were included in the synthesis. Chatbot-based risk assessment showed adequate response alignment with expert judgment at the extremes of suicide risk, but consistently failed to distinguish intermediate risk levels across multiple model families. Across 29 tested commercial chatbot agents, none met the criteria for an adequate suicidal crisis response. A clinically designed, framework-anchored chatbot achieved high efficacy across six outcome domains. Three percent of social chatbot users reported halted suicidal ideation, and a purpose-built clinical chatbot in emergency department settings significantly improved evidence-based care delivery. Systematic risk severity underestimation and the absence of cross-cultural evaluation were identified across all studies. AI-driven chatbots show potential as adjunctive tools across the suicide care continuum. Clinically designed, evidence-anchored chatbots demonstrate feasibility and meaningful benefit; commercially deployed chatbots without clinical validation demonstrate near-universal crisis response inadequacy. Mandatory clinical validation prior to public release, clinician oversight, and crisis system integration are prerequisites for responsible deployment.

## Impact statement

Suicide is among the leading causes of preventable death globally, affecting all age groups and disproportionately impacting young people. Conventional clinical risk assessment is constrained by workforce availability, subjective clinical judgment, and limited scalability. This narrative review evaluates AI-driven chatbots as adjunctive tools across the suicide care continuum, examining the evidence for their performance in risk assessment, intervention delivery, and ethical deployment. What we found reflects a clearly tiered performance landscape. Clinically designed, framework-anchored chatbots demonstrate feasibility and meaningful efficacy in suicide risk assessment and crisis intervention. In contrast, commercially deployed AI chatbots without clinical validation demonstrated systematic risk miscalibration, a consistent failure to distinguish intermediate risk levels, and near-universal inadequacy in crisis response, with none of 29 tested commercial apps meeting criteria for an adequate response. The principal determinant of safe and effective chatbot performance in suicide care is clinical intentionality in design, not the underlying AI technology. By synthesizing evidence from 11 primary studies spanning 2021–2025, including four newly published studies identified during the revision period, this review provides the most current synthesis of chatbot performance across the full suicide care continuum. Three critical gaps are identified as requiring urgent attention: the absence of standardized performance benchmarks for chatbot suicide assessment, the lack of mandatory pre-deployment clinical validation, and insufficient cross-cultural evidence. These findings carry direct implications for clinicians, policymakers, regulators, and technology developers. The deployment of AI-driven chatbots in mental health contexts without rigorous clinical validation poses documented harms, including the active provision of lethality-enabling information. Responsible deployment requires clinical framework grounding, mandatory independent validation, clinician oversight, and crisis service integration before chatbots can be considered safe adjuncts to suicide prevention.

## Introduction

Suicide is a significant public health concern and a leading cause of preventable death. In 2022, the age-adjusted suicide rate in the United States was 14.2 deaths per 100,000 population, representing a 5% increase from 2020. Suicide remains among the leading causes of death for individuals aged 10–34 (Garnett and Curtin, 2024). Additionally, 5.2% of adults aged 18 and older in the United States, or about 13.2 million people, reported having serious thoughts of suicide (Center for Behavioral Health Statistics [SAMSHA], 2024).

Assessing a suicidal patient is a highly sensitive task that requires an experienced clinician to combine clinical judgment with knowledge of previously established risks and protective factors, and in many cases, such decisions can be extremely challenging to make (Franklin et al., 2017). The use of traditional methods that rely solely on risk factors to predict suicidal behaviors has been shown to perform only slightly better than chance (Franklin et al., 2017). Even within well-characterized high-risk clinical populations such as those with major depressive disorder, a systematic review and meta-analysis found that risk stratification for suicidal ideation, attempts, and suicide death remains complex and multifactorial (Li et al., 2022). Thus, new techniques are being developed and tested for their ability to assess suicidal patients. Among the most clinically promising are AI-driven chatbots, conversational systems that engage users in real-time natural language dialog, distinct from passive LLM text classifiers that process data without any conversational interface.

Beyond chatbots, the use of artificial intelligence in suicide prevention has been extended to social media surveillance systems (Coppersmith et al., 2018), smartphone-based ecological momentary assessment (Kleiman et al., 2017), clinical decision support systems drawing on electronic health records (Walsh et al., 2017), and scalable digital interventions (Christensen et al., 2014). Computerized cognitive behavioral therapy for anxiety and depressive disorders has been shown to be effective, acceptable, and practical, with a mean effect size of *g* = 0.88 across 22 randomized controlled trials (Andrews et al., 2010), establishing the empirical foundation from which AI-driven chatbot interventions have evolved. Spanning from individual-level prediction analytics to population-level risk identification and clinical management, the application of AI to suicide care has been described as a potential means of identifying at-risk individuals, informing resource mobilization, and supporting clinical evaluation, medication management, and behavioral therapy delivery (Fonseka et al., 2019).

Growing scientific attention has focused on AI-driven chatbots for mental health applications, primarily depression management (Abd-alrazaq et al., 2019). However, their integration into healthcare raises concerns regarding data privacy, transparency, and inappropriate substitution of clinical judgment (Martinez-Martin et al., 2018). Despite the rapid proliferation of AI-driven chatbots in mental health settings, rigorous evaluation of their performance in suicide-specific contexts has remained limited, with emerging evidence suggesting both promise in clinically designed systems and substantial inadequacy in commercially deployed applications.

Despite these developments, no synthesis has evaluated AI-driven chatbots specifically across the full suicide care continuum. This narrative review aims to: evaluate the performance of AI-driven chatbots in detecting and assessing suicidal ideation relative to clinical benchmarks; examine the effectiveness of chatbot-based interventions; and identify ethical, cultural, and implementation challenges. The intervention boundary is explicitly defined as AI-driven chatbots, conversational systems engaging users in real-time dialog, excluding LLMs used purely as backend text classifiers.

## Methods

### Reporting framework

This review was conducted in accordance with the PRISMA 2020 guidelines (Page et al., 2021), adapted as a transparency framework for the structured database search and selection process, consistent with their adapted use in narrative reviews employing systematic searches (Mengist et al., 2020). The primary quality and reporting framework was SANRA (Baethge et al., 2019). The study was not prospectively registered, consistent with narrative review methodology.

### Search strategy and study selection

A comprehensive literature search was conducted through six electronic databases: PubMed (Medline), Cochrane Central Register of Controlled Trials (CENTRAL), Scopus, Embase, PsycINFO, and Web of Science (WOS). The initial search was conducted in December, 2024 with no lower date restriction. No upper cutoff date was applied; the literature was later updated through targeted literature monitoring, consistent with the narrative review approach of incorporating the most current available evidence. The complete PubMed search string was: ((Chatbot* OR ChatGPT OR GPT OR Gemini OR “Artificial Intelligence” OR AI OR Chatterbot OR Talkbot OR “Virtual Agent” OR “Virtual Assistant” OR LLM* OR “Large Language Model*”) AND (Suicide* OR “Suicidal thought*” OR “Suicidal act*” OR “Suicidal ideation*” OR “Suicidal risk*” OR Self-harm OR Self-injury)). Equivalent adaptations are provided in the Supplementary File.

### Population, intervention, comparison, and outcomes (PICO)

The studies included in this review were evaluated based on the PICO framework (Santos et al., 2007). The population of interest included individuals at risk of suicide, those experiencing suicidal ideation, or individuals with a history of self-harm or suicide attempts. The intervention was AI-driven chatbots, conversational systems that engage users or standardized user simulations in real-time natural language dialog, including clinically deployed purpose-built platforms (*e.g.*, Jaspr Health, SIMON, Replika) and general-purpose LLMs accessed *via* their conversational chatbot interfaces (*e.g.*, ChatGPT, Claude, and Gemini). Studies in which LLMs processed text as backend classifiers without any user-facing conversational interface were excluded from primary inclusion, as they do not constitute chatbot interventions. Comparison groups, where applicable, included human mental health professionals, care-as-usual interventions, or no comparator. Primary outcomes included chatbot performance in assessing and managing suicide risk, reductions in suicidal ideation, user engagement, and chatbot response accuracy relative to clinical benchmarks.

### Inclusion and exclusion criteria

#### Inclusion criteria

Studies were included if they: (1) involved AI-driven chatbots, conversational interfaces engaging users or standardized user simulations in real-time dialog, in the context of suicide risk assessment, prevention, or intervention; (2) enrolled or simulated populations at risk of suicide, suicidal ideation, or self-harm, or systematically evaluated chatbot responses to suicide-related queries using clinician-categorized risk frameworks; (3) presented primary data using experimental, observational, vignette-based, pilot RCT, content analysis, or query-response evaluation designs; (4) were published in peer-reviewed journals; and (5) were available in English as full texts. Studies addressing depression or loneliness as outcomes were included only when the target population had a documented relationship to suicidal risk.

#### Exclusion criteria

Studies were excluded if they employed LLMs or AI systems exclusively as backend text classifiers without any user-facing conversational interface, as this does not constitute a chatbot intervention; focused on rule-based or static decision-tree chatbots without adaptive natural language processing; addressed general AI applications without specific suicide-related outcomes; lacked primary data; were not published in peer-reviewed journals; or were published in languages other than English. Studies examining hybrid platforms in which rule-based components are embedded within AI-driven conversational architectures were retained.

#### Study selection process

Records retrieved from the database search were first added to EndNote to remove duplicates, and were then uploaded to Rayyan software to start the title and abstract screening (Ouzzani et al., 2016). Articles cleared during initial screening underwent full-text evaluation. Disagreements were resolved by discussion or consultation with the first author. Four additional primary studies published in 2025 were identified through targeted literature monitoring during the revision period and formally incorporated as included studies.

#### Data extraction and synthesis

A standardized data extraction form developed using Google Sheets was pilot tested before use. Three randomly selected studies were used to assess its clarity and consistency, and feedback was incorporated to refine the final version. Data were independently extracted by two researchers regarding study design, country of origin, chatbot system type and architecture, sample size, population demographics, interventions, outcomes, and limitations. Discrepancies were resolved by consensus; where unresolved, the first author was consulted.

#### Thematic narrative synthesis

Extracted data were synthesized using thematic narrative synthesis (Thomas and Harden, 2008), comprising: (1) free-line coding of individual study findings; (2) grouping of related codes into descriptive themes; and (3) development of analytical themes. Five final themes were agreed upon by all researchers: (1) chatbot performance in suicide risk assessment; (2) therapeutic intervention capabilities; (3) real-world deployment and effectiveness; (4) post-crisis monitoring and clinical integration; and (5) ethical considerations and potential harms.

#### Quality appraisal

Given the heterogeneity of the included studies, a narrative methodological appraisal was conducted rather than using formal risk-of-bias tool. Two researchers independently assessed each study across five domains: study design rigor, sample representativeness, chatbot validity, outcome measurement quality, and risk of bias. Each domain was rated as low, moderate, or high concern. Disagreements were resolved by discussion and by consultation with the first author.

## Results

### Search results and study selection

**Figure 1.**
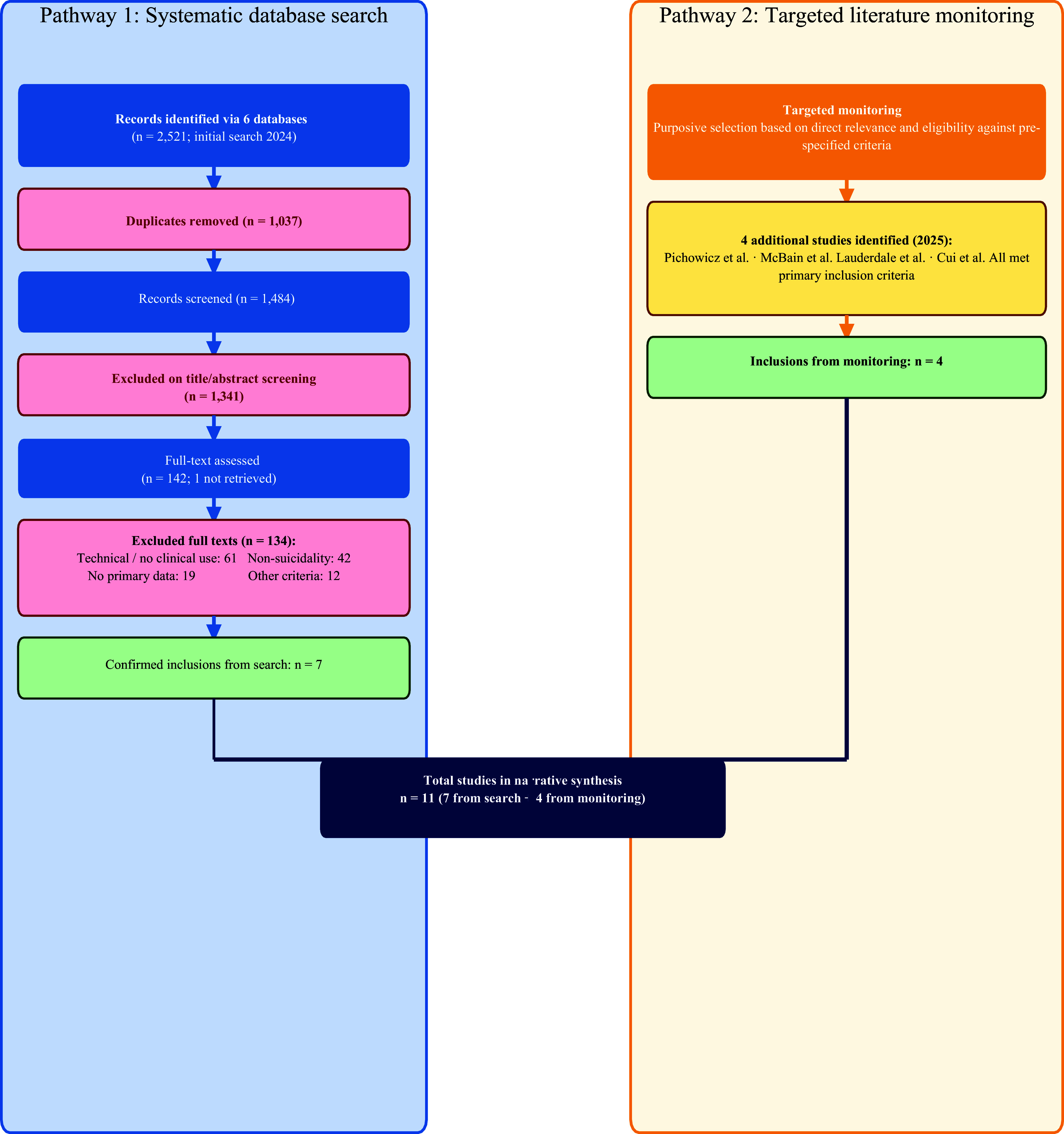
Literature identification process. Figure 1. long description.

The initial database search conducted in 2024 yielded 2,521 records across six databases. After the removal of 1,037 duplicate records, 1,484 records underwent title and abstract screening. Of these, 1,341 were excluded, leaving 143 records for full-text assessment. One full-text article could not be retrieved. Of the 134 excluded full-text articles, 61 (45.5%) were computational or technical design studies without clinical application; 42 (31.3%) addressed mental health conditions other than suicidality as standalone outcomes; 19 (14.2%) lacked primary data; and 12 (9.0%) did not meet population or design criteria. Seven studies met all the inclusion criteria from the initial search. Later, targeted literature monitoring identified four additional primary studies published in 2025 that met all eligibility criteria (Cui et al., 2025; Lauderdale et al., 2025; McBain et al., 2025; Pichowicz et al., 2025). The final primary study count was 11. The literature identification process is illustrated in Figure 1.

### Characteristics of included studies

The 11 included studies were published between 2021 and 2025 and were conducted in the United States (*n* = 4), Israel (*n* = 2), China (*n* = 1), Singapore (*n* = 1), Switzerland (*n* = 1), Poland (*n* = 1), and a multinational setting (*n* = 1). Study designs comprised one pilot RCT, one study protocol, two vignette studies, one brief research report, one comparative vignette study, one cross-sectional survey, one content analysis, one query-response evaluation, one app evaluation, and one LLM chatbot development and evaluation study. Chatbot systems examined included ChatGPT-3.5, ChatGPT-4, ChatGPT-4o, Claude 3.5 Sonnet, Gemini 1.5 Pro, Replika (GPT-3), Jaspr Health, SIMON, 29 commercial mental health chatbot agents, and a fine-tuned GPT-4 chatbot using the ACT framework. A visual summary of all included studies is presented in Table 1.

**Table 1.** Characteristics and key performance outcomes Table 1. long description.

Study	Chatbot/AI system	Country	Design	*N*	Key finding	Performance
Elyoseph and Levkovich (2023)	ChatGPT (Mar 14)	Israel	Brief research report	Simulated	High-risk cases at the 5th percentile; underestimation of suicide attempt risk in all vignette conditions	Underestimates
Levkovich and Elyoseph (2023)	ChatGPT–3.5 *vs.* GPT–4	Israel	Vignette study	Simulated	GPT–4 comparable to professionals for attempt probability; GPT–3.5 markedly lower; both underestimated resilience	GPT–4 better
Shinan-Altman et al. (2024)	ChatGPT–3.5 *vs.* GPT–4	Israel/UK	Vignette study	Simulated	GPT–4 detected depression × weapons interaction; GPT–3.5 failed to weigh lethal means access	GPT–4 better
Maples et al. (2024)	Replika (GPT–3)	USA	Cross-sectional survey	1,006	3% reported Replika halted SI; 90% lonely (43% severely or very severely); reduced anxiety; improved relationships	Modest (3%)
Martinengo et al. (2022)	9 commercial chatbots	Singapore	Content analysis	NR	Good CBT delivery and empathic tone; none provided adequate crisis response; personalization deficits	Moderate
Dimeff et al. (2021)	Jaspr Health (tablet app)	USA	Pilot RCT	31	Higher EBP delivery (*p* < 0.01); lower agitation; higher satisfaction *vs.* care as usual	Positive
Sels et al. (2021)	SIMON chatbot + passive sensing	Switzerland	Study protocol	100 (planned)	Text-based chatbot delivers 5×/day EMA combined with passive sensing to predict suicidal ideation and readmission post-discharge	Protocol only
Lauderdale et al. (2025)	ChatGPT–3.5, GPT–4o, Gemini	USA	Comparative vignette study	4 vignettes	Bidirectional miscalibration: most acute case underrated, least acute overrated *vs.* MHCPs; GPT–4o hospitalized all veterans regardless of acuity level	Miscalibrated
McBain et al. (2025)	ChatGPT–4o mini, Claude 3.5 Sonnet, Gemini 1.5 Pro	USA	Query-response evaluation (*N* = 9,000)	30 × 100	Aligned at extremes; failed to distinguish intermediate risk levels; Claude AOR = 2.01, Gemini AOR = 0.09 *vs.* ChatGPT; lethality info provided in some responses	Partial alignment
Pichowicz et al. (2025)	29 commercial chatbot agents	Poland	App evaluation (C-SSRS prompts)	29 apps	0% adequate; 51.72% marginal; 48.28% inadequate; no emergency contact info; no contextual understanding of escalating risk	Inadequate
Cui et al. (2025)	Fine-tuned GPT–4 (ACT framework)	China	Development + evaluation study	NR	High effectiveness on all 6 domains: interface operability, interaction experience, emotional support, intervention efficacy, safety/privacy, overall satisfaction	Positive

Figure 1. EBP: evidence-based practice; EMA: ecological momentary assessment; MHCPs: mental health care providers; NR: not reported; RCT: randomized controlled trial; SI: suicidal ideation; VHA: Veterans Health Administration.

### Quality appraisal summary

The pilot RCT (Dimeff et al., 2021) showed the most favorable methodological profile. Large-scale query-response evaluations (McBain et al., 2025) (*N* = 9,000) and the commercial chatbot evaluation (Pichowicz et al., 2025) (29 agents) showed low-to-moderate overall concern. Vignette-based studies raised high concern for sample representativeness due to their exclusive reliance on simulated cases. The cross-sectional survey raised high concern for bias (Maples et al., 2024). The SIMON study protocol (Sels et al., 2021) was not assessable for outcomes. A complete appraisal summary is presented in Table 2.

**Table 2.** Narrative methodological quality appraisal Table 2. long description.

Study	Study design rigor	Sample representativeness	Chatbot validity	Outcome measurement	Risk of bias	Overall
Elyoseph and Levkovich (2023)	High concern	High concern	Moderate concern	Moderate concern	High concern	High concern
Levkovich and Elyoseph (2023)	High concern	High concern	Moderate concern	Moderate concern	High concern	High concern
Shinan-Altman et al. (2024)	Moderate concern	High concern	Moderate concern	Moderate concern	Moderate concern	Moderate concern
Maples et al. (2024)	Moderate concern	Moderate concern	Moderate concern	High concern	High concern	High concern
Martinengo et al. (2022)	Moderate concern	High concern	Moderate concern	Moderate concern	Moderate concern	Moderate concern
Dimeff et al. (2021)	Low concern	Moderate concern	Low concern	Low concern	Moderate concern	Low concern
Sels et al. (2021)	N/A	N/A	Moderate concern	N/A	High concern	N/A
Lauderdale et al. (2025)	Moderate concern	High concern	Low concern	Moderate concern	Moderate concern	Moderate concern
McBain et al. (2025)	Low concern	Low concern	Low concern	Low concern	Moderate concern	Low concern
Pichowicz et al. (2025)	Low concern	Moderate concern	Low concern	Low concern	Moderate concern	Low concern
Cui et al. (2025)	Moderate concern	High concern	Low concern	Moderate concern	Moderate concern	Moderate concern

*Note:* “Chatbot validity” assesses whether the chatbot was deployed as a genuine conversational system. N/A: not assessable (protocol study). Concern levels – Low concern: no major methodological concerns; Moderate concern: some concerns unlikely to substantially alter findings; High concern: substantial concerns affecting validity.

**Figure 2.**
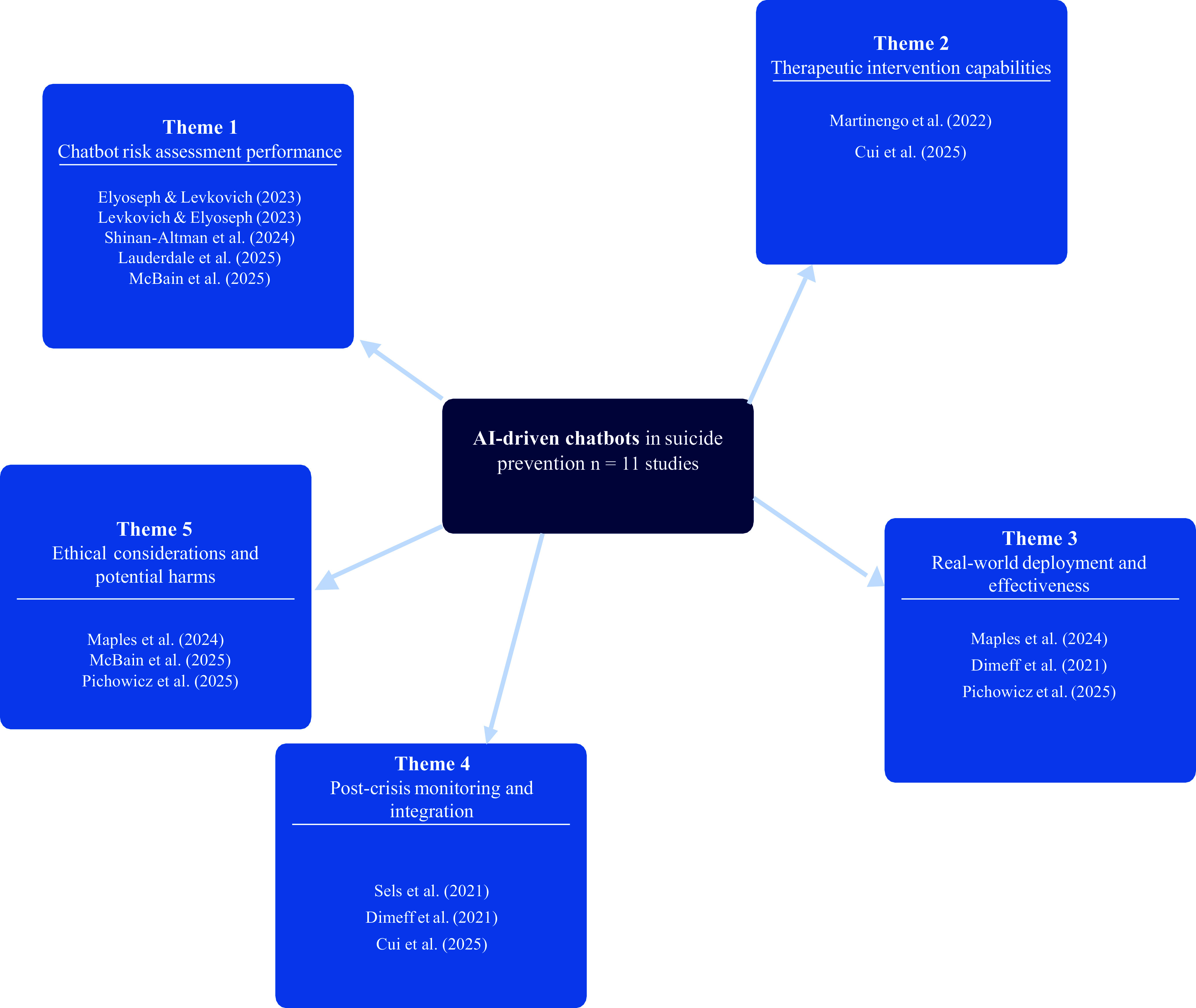
Thematic synthesis map. Figure 2. long description.

The synthesis identified five main themes. The thematic synthesis map is illustrated in Figure 2.

### Theme 1: Chatbot performance in suicide risk assessment

#### Chatbot response alignment with clinical benchmarks

Across four included studies evaluating chatbot risk assessment performance against clinical benchmarks, a consistent pattern emerged: adequate alignment at the extremes of risk, systematic failure at intermediate risk levels, and bidirectional calibration errors that raise acute clinical safety concerns.

Elyoseph and Levkovich, (2023) found that the March 14 version of ChatGPT consistently underestimated suicide attempt risk, ranking high-risk cases in the lowest percentile (5th). (Lauderdale et al., 2025) corroborated this pattern in a veteran-specific context, finding bidirectional miscalibration: ChatGPT-3.5, GPT-4o, and Gemini collectively underrated the most acute cases and overrated the least acute, compared with mental health care providers using the VHA Risk Stratification Table. Treatment planning by these chatbots was predicted by chronic but not acute risk ratings. GPT-4o recommended hospitalization for all veterans regardless of acuity level. McBain et al. (2025) provided the most methodologically robust evidence to date, generating 9,000 responses from three commercial chatbots (ChatGPT-4o mini, Claude 3.5 Sonnet, and Gemini 1.5 Pro) across 30 expert-categorized queries repeated 100 times each. ChatGPT and Claude provided direct responses to very-low-risk queries 100% of the time, and all three chatbots declined to respond to very-high-risk queries, indicating appropriate alignment at the extremes. However, LLMs did not meaningfully distinguish intermediate risk levels: the odds of a direct response were not statistically different across low-, medium-, and high-risk queries compared to very-low-risk queries. Claude was significantly more likely to respond directly than ChatGPT (AOR = 2.01, 95% CI = 1.71−2.37, *p* < 0.001), while Gemini was significantly less likely (AOR = 0.09, 95% CI = 0.08−0.11, *p* < 0.001). Both ChatGPT and Claude provided direct answers to lethality-enabling questions about firearms and poisons.

#### Comparative performance between chatbot generations and risk factor recognition

Levkovich and Elyoseph, (2023) found that ChatGPT-3.5 was below professionals in predicting suicide risk, while ChatGPT-4 performed similarly to professionals in estimating the probability of a suicide attempt but exaggerated suicidal ideation and psychache. Shinan-Altman et al. (2024) found that ChatGPT-4 adequately identified the interaction between depression history and access to weapons, while ChatGPT-3.5 did not. Across five included studies evaluating chatbot risk rating accuracy, Shinan-Altman et al. (2024) found that ChatGPT-4 adequately identified the interaction between depression history and access to weapons, while ChatGPT-3.5 did not.

### Theme 2: Therapeutic intervention capabilities

#### Chatbot-based therapeutic support

Martinengo et al. (2022) conducted a content analysis of nine commercial chatbots and found that conversational agents provided anonymous, empathic interaction and were effective at conducting structured psychotherapeutic exercises, yet were inadequate for comprehensive suicide risk management and had difficulty with personalization. None provided an adequate response to high-risk crisis scenarios. By contrast, Cui et al. (2025) developed and evaluated a clinically designed chatbot, fine-tuning GPT-4 through structured prompt engineering based on the ACT model (Assessment–Crisis Intervention–Trauma Treatment) derived from validated psychological crisis intervention frameworks. The system was implemented as a self-help web-based dialog platform and evaluated across six dimensions: user interface operability, interaction experience, emotional support quality, intervention efficacy, safety and privacy, and overall satisfaction. High effectiveness was achieved across all six domains, representing the first study in this review to demonstrate that a clinically anchored chatbot can achieve high intervention quality for users experiencing suicidal ideation.

### Theme 3: Real-world deployment and effectiveness

#### Real-world chatbot effectiveness

Maples et al. (2024) found that among 1,006 Replika chatbot student users, 90% were lonely (43% severely or very severely lonely) and 3% specifically reported that Replika halted active suicidal ideation. Additional outcomes included reduced anxiety and improved self-reported quality of interpersonal relationships.

#### Evidence-based clinical chatbot deployment

Dimeff et al. (2021) conducted a pilot RCT and concluded that the Jaspr Health tablet application for delivering evidence-based suicide prevention in emergency departments was feasible, acceptable, and effective. The intervention group received significantly more evidence-based care components than control participants (*p* < 0.01), with lower agitation scores and greater satisfaction ratings. Pichowicz et al. (2025) evaluated 29 commercially available AI-powered chatbot apps against standardized C-SSRS-based simulated crisis prompts. None met the pre-specified criteria for an adequate response; 51.72% met only relaxed marginal criteria, and 48.28% were fully inadequate. The most common failure modes were the inability to provide emergency contact information and the lack of contextual understanding of escalating suicidal risk.

### Theme 4: Post-crisis monitoring and clinical integration

#### Post-discharge chatbot monitoring

Sels et al.’s (2021) SIMON protocol combined SIMON-SELF (a text-based chatbot delivering ecological momentary assessment five times daily) and SIMON-SENSE (passive behavioral sensing) in 100 psychiatric patients during the 4 week period following hospital discharge to predict suicidal ideation and readmission.

#### Integration with crisis services

Of the 11 included studies, only Dimeff et al. (2022) explicitly designed a chatbot for integration within an existing clinical workflow. Pichowicz et al. (2025) found that the failure to provide emergency contact information, the most fundamental crisis system linkage requirement, was among the most common deficiencies across 29 commercially deployed chatbots, confirming that real-world app deployment has not addressed even basic crisis integration requirements.

### Theme 5: Ethical considerations and potential harms

#### Dependence concerns

Maples et al.’s (2024) concern about reliance on AI for mental healthcare was raised in a context where 90% of users reported loneliness and 43% reported severe loneliness, suggesting pre-existing social isolation that may heighten dependency risk.

#### Safety risks and data privacy

McBain et al. (2025) identified a specific chatbot-mediated lethality information risk: both ChatGPT and Claude provided direct responses to questions about which firearms, poisons, or methods had the highest association with completed suicide. Pichowicz et al. (2025) corroborated this finding by demonstrating inadequate crisis response dimension, with 48.28% of tested commercial chatbot apps failing entirely to redirect suicidal users toward emergency services.

## Discussion

### Overview

This narrative review synthesized findings from 11 studies (2021–2025) evaluating AI-driven chatbots across the suicide care continuum. A three-tier performance landscape emerges clearly: (1) clinically designed, evidence-anchored chatbots show feasibility and meaningful efficacy; (2) general-purpose commercial chatbots used for risk assessment show systematic intermediate-risk calibration failure and lethality information safety risks; and (3) commercially deployed mental health apps without clinical validation demonstrate near-universal crisis response inadequacy. The primary determinant of safe and effective chatbot performance in suicide care is clinical intentionality in design, not the underlying AI technology.

### Chatbot performance in suicide risk assessment: Concordance and discordance with the literature

The finding that chatbots demonstrate adequate response alignment at the extremes of suicide risk but systematic failure at intermediate levels (McBain et al., 2025) is consistent with the broader challenge of suicidal behavior prediction documented by Franklin et al. (2017), who found that across 50 years of research that predictive accuracy across all methods remains only marginally above chance. (Belsher et al., 2019), in a systematic review of 17 machine learning suicide prediction models, found that while global classification accuracy was good (AUC ≥0.80 in most models), the positive predictive validity for suicide mortality was extremely low (PPV ≤0.01 in most models), such that a positive prediction result carried near-zero clinical utility, a structural limitation mirrored in the intermediate-risk failure observed across chatbots. Carter et al. (2017) found that even established clinical risk scales show pooled positive predictive values as low as 5.5%, contextualizing ChatGPT’s underestimation within a broader historical challenge.

The bidirectional miscalibration observed by Lauderdale et al. (2025) – underrating the most acute cases and overrating the least acute – extends beyond earlier single-direction underestimation findings (Elyoseph and Levkovich, 2023) and suggests that chatbots are applying population-level base-rate priors that compress risk estimates toward the mean, a regression-to-the-mean artifact. This is particularly dangerous at the acute high end of the risk spectrum: treatment planning by these chatbots was predicted by chronic but not acute risk ratings, whereas acute risk is the primary driver of emergency intervention decisions. GPT-4o’s recommendation of hospitalization for all veterans regardless of acuity level further illustrates an overcautious failure mode that would generate resource overutilization in real-world deployment. The finding by McBain et al. (2025) that intermediate-risk queries received no statistically different response rates than low-risk queries (despite expert consensus that they required different handling) confirms that current commercial chatbots lack the risk differentiation capability that is clinically essential for safe crisis management. Furthermore, the provision of direct answers to lethality-enabling questions about firearms and poisons by both ChatGPT and Claude represents a distinct safety risk beyond classification failure alone.

Thomas et al. (2025) provide complementary evidence from a non-chatbot context: using the Mixtral LLM to rate 100 real youth crisis helpline transcripts against NGASR expert ratings, they found that zero-shot prompting at temperature 0 yielded perfect inter-rating reliability (*α* = 1.00), while few-shot prompting showed best human-AI agreement for very high risk (*α* = 0.78). Critical clinical items showed poor validity regardless of configuration. Although Mixtral was employed as a backend classifier rather than a chatbot in this study, the finding that clinical item-level validity remains poor even under optimal LLM configurations has direct implications for the capabilities of deployed chatbots, whose underlying models share these structural limitations. Across all five included studies evaluating chatbot risk rating accuracy, no single chatbot model consistently matched clinician performance across all evaluated dimensions, and substantial inter-model variability was documented. This argues against the assumption that any commercially available chatbot can serve as a reliable clinical risk assessment tool without domain-specific clinical design.

### Evaluating chatbot efficacy: Outcome heterogeneity and standards

A critical cross-cutting observation is the substantial heterogeneity in how chatbot effectiveness was operationalized across the 11 included studies: Likert-scale risk ratings against professional norms (Elyoseph and Levkovich, 2023; Levkovich and Elyoseph, 2023; Shinan-Altman et al., 2024); VHA risk stratification alignment (Lauderdale et al., 2025); direct response likelihood categorized by risk level (McBain et al., 2025); pre-specified adequacy criteria (Pichowicz et al., 2025); evidence-based care delivery rates (Dimeff et al., 2021); multi-domain efficacy ratings (Cui et al., 2025); self-reported ideation cessation (Maples et al., 2024); and content quality coding (Martinengo et al., 2022). This eight-framework heterogeneity across 11 studies precludes quantitative synthesis and reflects the absence of a consensus minimum performance standard for chatbot suicide assessment tools. The field urgently requires a standardized benchmark framework analogous to the C-SSRS (Posner et al., 2011), adapted for evaluating chatbot performance across the suicide care continuum.

### Chatbot-based clinical interventions and the design–efficacy relationship

Martinengo et al. (2022) demonstrated adequate CBT exercise delivery but inadequate crisis response in nine commercial chatbots. Firth et al.’s (2017) meta-analysis of 18 smartphone intervention RCTs (*N* = 3,414) reported significant but modest reductions in depressive symptoms (Hedges’ *g* = 0.38), contextualizing the therapeutic effect sizes observed across chatbot studies. Critically, a meta-analysis of 19 RCTs of standalone mental health apps found no significant pooled effect on suicidal ideation or self-injury (Hedges’ *g* ranging from −0.14 to 0.18 across these outcomes), even while modest effects on depression were observed (*g* = 0.33), underscoring the difficulty of achieving suicidality-specific outcomes through unguided digital tools and reinforcing why clinical framework integration is essential (Weisel et al., 2019). The design–efficacy relationship is now clearly established across the 11 included studies. Chatbots explicitly designed around clinical frameworks, including Jaspr Health (CAMS model; (Dimeff et al., 2021) and the (Cui et al., 2025) chatbot (ACT framework), both demonstrated high efficacy outcomes in their respective evaluation contexts. Chatbots deployed without clinical framework grounding, including the 29 commercial apps in Pichowicz et al. (2025) and the 9 apps in Martinengo et al. (2022) demonstrated inadequate or moderate performance. This dose–response relationship between clinical design and chatbot efficacy is the strongest practical conclusion of this review. The key differentiator between clinically anchored and commercially deployed chatbots is clinical design: ACT framework-anchored prompt engineering, as demonstrated by Cui et al. (2025), produced substantially better crisis-relevant performance than general-purpose chatbot deployment, while Jaspr Health was purpose-built around the CAMS theoretical framework with clinical validation, in contrast to the 29 apps evaluated by Pichowicz et al. (2025), which were general mental distress apps without crisis-specific clinical validation.

Moylan and Doherty’s (2025) expert analysis of Wysa and Replika from a mental health professional perspective confirms this from the clinician side: professionals identified generic modes of care, dependency-promoting interaction patterns, and harm-inviting response designs as primary concerns, underscoring that clinical practitioner perspectives should be systematically integrated into chatbot development processes.

### Real-world deployment and population-level reach

Maples et al. (2024) found that 3% of Replika users reported halted suicidal ideation. These findings suggest that chatbots may operate primarily through indirect pathways, alleviating loneliness and supporting emotional regulation, rather than direct crisis intervention, consistent with Fitzpatrick et al.’s (2017) RCT of Woebot demonstrating moderate and significant depression symptom reductions (*d* = 0.44) at 2 weeks. Qualitative evidence from 19 real-world users of generative AI chatbots for mental health corroborates both the potential and the limitations identified in clinical evaluations: users reported meaningful benefits, including a sense of emotional sanctuary and personal insight into relationships, while simultaneously identifying the urgent need for stronger safety guardrails and more persistent clinical integration (Siddals et al., 2024). Pichowicz et al.’s (2025) finding that none of the 29 commercially available chatbot agents provided an adequate crisis response contextualizes these modest benefits within a larger landscape of inadequate real-world deployment. The contrast between Dimeff et al.’s (2021) findings and those of Pichowicz et al. (2025) is central to understanding the chatbot performance landscape; it confirms that chatbot efficacy is not a property of the underlying technology but rather of clinical intentionality in design. This inadequacy has historical precedent; a 2016 systematic evaluation of 49 interactive suicide prevention apps found that only 26.5% contained at least one WHO-endorsed evidence-based strategy, none provided comprehensive evidence-based support, and potentially harmful content was identified in a subset of apps (Larsen et al., 2016), suggesting that the commercial app ecosystem has made limited progress in nearly a decade. The implication is that the 3% direct benefit found by Maples et al. (2024) may represent an upper bound for commercially deployed chatbots lacking clinical validation, not a baseline.

### Post-crisis monitoring and system integration

The SIMON protocol (Sels et al., 2021) addresses the post-discharge high-risk transition documented by Chung et al. (2019), who found pooled suicide rates of 2,950 per 100,000 person-years in the first week post-discharge, confirming that this period represents the highest-risk window in psychiatric care. Episodic routine follow-up represents a primary limitation of current post-discharge care, and this is precisely the gap that SIMON’s continuous chatbot monitoring is designed to address. Cui et al.’s (2025) self-help platform represents a scalable complement to SIMON’s monitoring model. The (Cui et al., 2025) self-help web chatbot platform offers a complementary model for post-crisis engagement, providing accessible intervention delivery that does not depend on clinical infrastructure; this is a critical advantage given McBain et al.’s (2025) observation that the United States has approximately one psychiatrist per 13,492 residents, addressing the scalability limitations that have constrained prior monitoring approaches. Of the 11 included studies, only Dimeff et al. (2021) designed a chatbot for integration within an existing clinical workflow, and Pichowicz et al. (2025) confirmed that crisis service linkage failures are the most common deficiency across commercially deployed apps. The disconnect between technology development and crisis service infrastructure is clearly reflected in the current evidence base. This pattern is consistent with previously identified fundamental misconceptions in how digital mental health technologies are conceptualized, developed, and integrated into care systems, including treating technology as a finished product rather than a service, and paying insufficient attention to user engagement and clinical workflow integration (Mohr et al., 2017).

### Toward clinically grounded chatbot development: Recommendations for training and design

A critical gap across the included studies is the absence of a clinical framework grounding in most evaluated chatbots. The systematic calibration failures observed across risk assessment studies reflect training data skewed toward general text corpora, where extreme-risk presentations are statistically rare, producing regression-to-the-mean errors at precisely the clinically critical high-risk end. Cui et al.’s (2025) demonstration that GPT-4, when fine-tuned through structured ACT framework prompt engineering, achieved high intervention quality without new clinical training data, provides an immediately actionable pathway: clinical framework-guided prompt engineering can substantially improve chatbot performance in the near term while clinically curated training datasets are developed. Future chatbot training should incorporate: (1) curated clinical datasets derived from validated structured clinical instruments (C-SSRS, CAMS) with deliberate oversampling of high-risk presentations; (2) passive biometric sensing and ecological momentary assessment combined with structured clinical data (Bourla et al., 2018; Sels et al., 2021); and (3) culturally diverse training data. All 11 included studies are from high-income countries, with only (Cui et al., 2025) representing a non-Western context.

### Potential harms, ethical considerations, and governance

The ethical risk profile of chatbots in suicide care is now two-sided. McBain et al. (2025) established that current commercial chatbots can provide lethality-enabling information under some query conditions, while Pichowicz et al. (2025) established that they simultaneously fail to provide adequate crisis redirection. Emerging from 9,000 standardized responses, the McBain et al. (2025) findings establish that current commercial chatbots can facilitate harm not merely through inadequate crisis response but through the active provision of lethality-enabling information. This bidirectional risk – potential harm facilitation and inadequate harm prevention – has no analog in prior human clinical practice and is not addressed by any currently existing regulatory framework for AI mental health tools. Moylan and Doherty’s (2025) finding that mental health professionals identified medium-to-low trust in commercial chatbots and multiple harm vectors (generic care, dependency risk, and manipulation potential) reinforces the urgent need for domain-specific governance frameworks developed in partnership with clinical experts. Prior evidence indicates that depression severity is consistently associated with problematic smartphone use, with at least medium effect sizes across reviewed studies (Elhai et al., 2017), suggesting a pre-existing vulnerability profile among chatbot users that amplifies dependency risks. The (Herbener and Damholdt, 2025) cross-sectional study of 1,599 Danish high school students corroborates the dependency risk profile identified by Maples et al. (2024), demonstrating that socially disconnected young people are disproportionately drawn to chatbots as emotional coping tools; social-supportive chatbot users reported significantly more loneliness (*d* = 0.53) and less perceived social support (*d* = 0.46) than non-chatbot users.

Campbell et al.’s (2025) longitudinal content analysis of seven chatbots across two phases (spring 2023 and summer 2024) found measurable improvement in the depth and accuracy of suicide-related responses over 1 year, with increased emphasis on the 988 crisis hotline and more comprehensive information about suicide warning signs. However, Microsoft Copilot provided an unsolicited image depicting suicide in Phase 1, illustrating that even improving AI systems remain capable of harmful outputs. This temporal evidence suggests that chatbot safety in suicide contexts is improving but remains uneven, and requires periodic independent review rather than one-time evaluation.

### Limitations

This narrative review has several limitations. The 11 included studies are exclusively from high-income countries, limiting generalizability to low- and middle-income countries and non-Western populations. Cross-cultural research has demonstrated that suicidal behavior and its communicative function differ meaningfully between Western and non-Western contexts. For instance, the four-factor semiotic model empirically supported in Western populations was not applicable in Uganda, where suicidal behavior followed a distinct two-factor communicative structure (Hjelmeland et al., 2008), suggesting that chatbot systems trained on Western clinical data may not perform equitably across cultural settings. The narrative review design carries inherent risks of selection bias. Outcome heterogeneity precluded quantitative synthesis. Publication bias cannot be excluded.

## Conclusion

AI-driven chatbots demonstrate a clearly tiered performance landscape in suicide care. Clinically designed, evidence-anchored chatbots show feasibility and meaningful efficacy; general-purpose commercial chatbots show systematic intermediate-risk calibration failure and chatbot-mediated lethality information risks; and commercially deployed mental health apps without clinical validation demonstrate near-universal crisis response inadequacy. The central conclusion is that chatbot efficacy in suicide care is determined not by the underlying AI technology but by clinical intentionality in design, framework grounding, and mandatory pre-deployment clinical validation. Clinician oversight, crisis system integration, culturally diverse training data, and standardized performance benchmarks are the essential conditions for responsible and beneficial deployment of AI-driven chatbots in suicide prevention.

## Supporting information

10.1017/gmh.2026.10260.sm001 Elbarbary et al. supplementary material Elbarbary et al. supplementary material

## Data Availability

Data are available within the manuscript and the Supplementary File.

## Supplementary material

The supplementary material for this article can be found at http://doi.org/10.1017/gmh.2026.10260.

## Long descriptions

### Figure 1. Long description

The flowchart is divided into two vertical panels that converge at the bottom.

Pathway 1: Systematic database search (Left Panel, Blue Background)

1. Records identified via 6 databases (n = 2,521; initial search 2024).

2. Duplicates removed (n = 1,037).

3. Records screened (n = 1,484).

4. Excluded on title/abstract screening (n = 1,341).

5. Full-text assessed (n = 142; 1 not retrieved).

6. Excluded full texts (n = 134). Reasons include: Technical / no clinical use: 61; Non-suicidality: 42; No primary data: 19; Other criteria: 12.

7. Confirmed inclusions from search: n = 7.

Pathway 2: Targeted literature monitoring (Right Panel, Yellow Background)

1. Targeted monitoring: Purposive selection based on direct relevance and eligibility against pre-specified criteria.

2. 4 additional studies identified (2025): Pichowicz et al., McBain et al., Lauderdale et al., Cui et al. All met primary inclusion criteria.

3. Inclusions from monitoring: n = 4.

Synthesis (Bottom Center)

Arrows from both pathways point to a final dark blue box: Total studies in narrative synthesis n = 11 (7 from search + 4 from monitoring).

Navigate back to Figure 1..

### Table 1. Long description

The table consists of seven columns: Study, Chatbot/A I system, Country, Design, N, Key finding, and Performance.

* Elyoseph and Levkovich 2023: ChatGPT Mar 14, Israel. Simulated study found underestimation of suicide attempt risk in all conditions. Performance: Underestimates.

* Levkovich and Elyoseph 2023: ChatGPT-3.5 vs. G P T-4, Israel. G P T-4 comparable to professionals; G P T-3.5 markedly lower. Performance: G P T-4 better.

* Shinan-Altman et al. 2024: ChatGPT-3.5 vs. G P T-4, Israel/U K. G P T-4 detected depression and weapons interaction; G P T-3.5 failed. Performance: G P T-4 better.

* Maples et al. 2024: Replika G P T-3, U S A. 3 percent reported Replika halted S I; 90 percent lonely. Performance: Modest 3 percent.

* Martinengo et al. 2022: 9 commercial chatbots, Singapore. Good C B T delivery but inadequate crisis response. Performance: Moderate.

* Dimeff et al. 2021: Jaspr Health, U S A. Higher E B P delivery p less than 0.01 and lower agitation. Performance: Positive.

* Sels et al. 2021: S I M O N chatbot, Switzerland. Protocol for predicting S I and readmission. Performance: Protocol only.

* Lauderdale et al. 2025: ChatGPT-3.5, G P T-4o, Gemini, U S A. Bidirectional miscalibration; G P T-4o hospitalized all veterans regardless of acuity. Performance: Miscalibrated.

* McBain et al. 2025: ChatGPT-4o mini, Claude 3.5 Sonnet, Gemini 1.5 Pro, U S A. Aligned at extremes but failed intermediate risk levels. Performance: Partial alignment.

* Pichowicz et al. 2025: 29 commercial agents, Poland. 0 percent adequate; no emergency contact info. Performance: Inadequate.

* Cui et al. 2025: Fine-tuned G P T-4 A C T framework, China. High effectiveness across 6 domains including emotional support and safety. Performance: Positive.

Navigate back to Table 1..

### Table 2. Long description

The table consists of seven columns: Study, Study design rigor, Sample representativeness, Chatbot validity, Outcome measurement, Risk of bias, and Overall.

* Elyoseph and Levkovich 2023: High concern for design, sample, bias, and overall; Moderate concern for validity and outcome.

* Levkovich and Elyoseph 2023: High concern for design, sample, bias, and overall; Moderate concern for validity and outcome.

* Shinan-Altman et al. 2024: Moderate concern for design, validity, outcome, bias, and overall; High concern for sample.

* Maples et al. 2024: Moderate concern for design, sample, and validity; High concern for outcome, bias, and overall.

* Martinengo et al. 2022: Moderate concern for design, validity, outcome, bias, and overall; High concern for sample.

* Dimeff et al. 2021: Low concern for design, validity, outcome, and overall; Moderate concern for sample and bias.

* Sels et al. 2021: N / A for design, sample, outcome, and overall; Moderate concern for validity; High concern for bias.

* Lauderdale et al. 2025: Moderate concern for design, outcome, bias, and overall; High concern for sample; Low concern for validity.

* McBain et al. 2025: Low concern for design, sample, validity, outcome, and overall; Moderate concern for bias.

* Pichowicz et al. 2025: Low concern for design, validity, outcome, and overall; Moderate concern for sample and bias.

* Cui et al. 2025: Moderate concern for design, outcome, bias, and overall; High concern for sample; Low concern for validity.

Navigate back to Table 2..

### Figure 2. Long description

At the center is a dark blue rectangular node labeled A I-driven chatbots in suicide prevention n = 11 studies. Five light blue arrows point outward to five peripheral boxes representing research themes.

* Theme 1 at the top-left is Chatbot risk assessment performance. It lists Elyoseph & Levkovich (2023), Levkovich & Elyoseph (2023), Shinan-Altman et al. (2024), Lauderdale et al. (2025), and McBain et al. (2025).

* Theme 2 at the top-right is Therapeutic intervention capabilities. It lists Martinengo et al. (2022) and Cui et al. (2025).

* Theme 3 at the center-right is Real-world deployment and effectiveness. It lists Maples et al. (2024), Dimeff et al. (2021), and Pichowicz et al. (2025).

* Theme 4 at the bottom-center is Post-crisis monitoring and integration. It lists Sels et al. (2021), Dimeff et al. (2021), and Cui et al. (2025).

* Theme 5 at the center-left is Ethical considerations and potential harms. It lists Maples et al. (2024), McBain et al. (2025), and Pichowicz et al. (2025).

Navigate back to Figure 2..
