# Supplementary material for: Are artificial intelligence chatbots safe for suicide risk assessment? A narratively synthesized review of current evidence
Source: Glob Ment Health (Camb). 2026 Jun 25;13:e135. doi: 10.1017/gmh.2026.10260 (PMC13373273; doi:10.1017/gmh.2026.10260)
Supplement: Elbarbary et al. supplementary material [file S205442512610260Xsup001.docx]

**Search Strategy**

**PubMed**

**Search scope:** Title/Abstract

((Chatbot*[tiab] OR ChatGPT[tiab] OR GPT[tiab] OR Gemini[tiab] OR "Artificial Intelligence"[tiab] OR AI[tiab] OR Chatterbot[tiab] OR Talkbot[tiab] OR "Virtual Agent"[tiab] OR "Virtual Assistant"[tiab] OR LLM*[tiab] OR "Large Language Model*"[tiab]) AND (Suicide*[tiab] OR "Suicidal thought*"[tiab] OR "Suicidal act*"[tiab] OR "Suicidal ideation*"[tiab] OR "Suicidal risk*"[tiab] OR Self-harm[tiab] OR Self-injury[tiab]))

**Embase**

**Search scope:** Title/Abstract/Emtree Terms

((Chatbot*:ti,ab,kw OR ChatGPT:ti,ab,kw OR GPT:ti,ab,kw OR Gemini:ti,ab,kw OR "Artificial Intelligence":ti,ab,kw OR AI:ti,ab,kw OR Chatterbot:ti,ab,kw OR Talkbot:ti,ab,kw OR "Virtual Agent":ti,ab,kw OR "Virtual Assistant":ti,ab,kw OR LLM*:ti,ab,kw OR "Large Language Model*":ti,ab,kw) AND (Suicide*:ti,ab,kw OR "Suicidal thought*":ti,ab,kw OR "Suicidal act*":ti,ab,kw OR "Suicidal ideation*":ti,ab,kw OR "Suicidal risk*":ti,ab,kw OR Self-harm:ti,ab,kw OR Self-injury:ti,ab,kw))

**PsycINFO**

**Search scope:** Title/Abstract/Thesaurus of Psychological Index Terms

((TI (Chatbot* OR ChatGPT OR GPT OR Gemini OR "Artificial Intelligence" OR AI OR Chatterbot OR Talkbot OR "Virtual Agent" OR "Virtual Assistant" OR LLM* OR "Large Language Model*") OR AB (Chatbot* OR ChatGPT OR GPT OR Gemini OR "Artificial Intelligence" OR AI OR Chatterbot OR Talkbot OR "Virtual Agent" OR "Virtual Assistant" OR LLM* OR "Large Language Model*") OR KW (Chatbot* OR ChatGPT OR GPT OR Gemini OR "Artificial Intelligence" OR AI OR Chatterbot OR Talkbot OR "Virtual Agent" OR "Virtual Assistant" OR LLM* OR "Large Language Model*")) AND (TI (Suicide* OR "Suicidal thought*" OR "Suicidal act*" OR "Suicidal ideation*" OR "Suicidal risk*" OR Self-harm OR Self-injury) OR AB (Suicide* OR "Suicidal thought*" OR "Suicidal act*" OR "Suicidal ideation*" OR "Suicidal risk*" OR Self-harm OR Self-injury) OR KW (Suicide* OR "Suicidal thought*" OR "Suicidal act*" OR "Suicidal ideation*" OR "Suicidal risk*" OR Self-harm OR Self-injury)))

**Scopus**

**Search scope:** Title/Abstract/Keyword

TITLE-ABS-KEY((Chatbot* OR ChatGPT OR GPT OR Gemini OR "Artificial Intelligence" OR AI OR Chatterbot OR Talkbot OR "Virtual Agent" OR "Virtual Assistant" OR LLM* OR "Large Language Model*") AND (Suicide* OR "Suicidal thought*" OR "Suicidal act*" OR "Suicidal ideation*" OR "Suicidal risk*" OR Self-harm OR Self-injury))

**Cochrane Central Register of Controlled Trials (CENTRAL)**

**Search scope:** Title/Abstract/Keyword

((Chatbot* OR ChatGPT OR GPT OR Gemini OR Artificial NEXT Intelligence OR AI OR Chatterbot OR Talkbot OR Virtual NEXT Agent OR Virtual NEXT Assistant OR LLM* OR Large NEXT Language NEXT Model*):ti,ab,kw AND (Suicide* OR Suicidal NEXT thought* OR Suicidal NEXT act* OR Suicidal NEXT ideation* OR Suicidal NEXT risk* OR Self-harm OR Self-injury):ti,ab,kw)

**Web of Science (WOS)**

**Search scope:** Topic

TS=((Chatbot* OR ChatGPT OR GPT OR Gemini OR "Artificial Intelligence" OR AI OR Chatterbot OR Talkbot OR "Virtual Agent" OR "Virtual Assistant" OR LLM* OR "Large Language Model*") AND (Suicide* OR "Suicidal thought*" OR "Suicidal act*" OR "Suicidal ideation*" OR "Suicidal risk*" OR Self-harm OR Self-injury))
