# Supplementary material for: Are artificial intelligence chatbots safe for suicide risk assessment? A narratively synthesized review of current evidence
Source: Glob Ment Health (Camb). 2026 Jun 25;13:e135. doi: 10.1017/gmh.2026.10260 (PMC13373273; doi:10.1017/gmh.2026.10260)
Supplement: Peer review history

# Reviewer report: Review: Are artificial intelligence chatbots safe for suicide risk assessment? A narratively synthesized review of current evidence — R0/PR2

**DOI:** 10.1017/gmh.2026.10260.pr2

**Authors:** Mohsen Khosravi

**Affiliations:** Shiraz University of Medical Sciences, Iran (the Islamic Republic of)

Greetings

My comments are presented below:

• Provide the main aim of the study in the abstract in a clear, precise and short format.

• Provide the year of conduction of the study in the abstract.

• Provide the time-span of the inclusion criteria in the abstract.

• Provide the analytical approach in the abstract.

• Provide the number of the included studies in the abstract.

• Provide the results specific to the main research question in the abstract.

• The conclusion in the abstract should not provide new data, not presented in the findings. Rather it should be an overall analysis of the findings.

• Present the findings of the manuscript in accordance to a formal guideline for data reporting (Ie. PRISMA), and cite it within the methodology section.

• Move the number of search hits to the results section

• Provide the main search keywords in the manuscript. in this regard, several important keywords are missed such as bot*, conversational agent*, natural language processing, etc. MeSH keywords are also missed. Moreover, suggest editing suicide* converting it to suicid* as the latter keyword would include suicidal keyword within the search results.

• Was your study limited to the large language models? Suggest adding the specialized chatbots in this regard, as LLMs are not specialized in risk assessment.

• Provide the inclusion/exclusion criteria in further details. Specify the criteria regarding research types(ie. RCTs, …), methodology approaches (qualitative or quantitative), … .

• Why rule-based chatbots are excluded?

• The PRISMA guideline is specified for data reporting, not for data collection. Kindly, move it to the initial part of the methodology section and adhere to the guideline throughout the manuscript.

• Move the data regarding the research hits, and final studies to the results section.

• Remove the phrase “a lot of reports”, as we don’t use such vague words in research manuscript. replace it with a precise quantitative data in this regard.

• Remove “many studies…”.

• Provide the data collection form and process.

• Provide the data analysis process in the methodology section.

• Provide details regarding measures to lower risk of bias and enhance study data validity and reliability.

• Present the study data in a structured format. Suggest u conducting thematic analysis adhering to a formal framework in this regard.

Sadly, the study lacks diverse necessary attributes to be considered suitable for publication. The research question is not precise. Several keywords are also missed. No data analysis has been conducted. The study also needs to get updated. In this regard, as an example, the study conducted by Pichowicz et al. in 2025 which has included 29 chatbots is missed due to the time-period of inclusion criteria in the study. Furthermore, suggest limiting the scope of the study to either large language models or the chatbots specified in suicide detection. Mixing up these two broad scopes would decrease readability and preciseness of the results.

With kind regards

# Reviewer report: Review: Are artificial intelligence chatbots safe for suicide risk assessment? A narratively synthesized review of current evidence — R0/PR3

**DOI:** 10.1017/gmh.2026.10260.pr3

This is a thoughtful and timely review of emerging research issues related to AI applications in mental health care, specifically related to suicide and suicidal ideation. A review such as this can provide a useful guide to readers interested in a general overview of research that has been conducted. At this point there are no firm conclusions that can be drawn from the small literature and the rapidly developing technology. Nonetheless, a review can be useful.

The review has some weaknesses that could be addressed. First, the boundaries of the review are unclear at times. Some of the papers that are discussed do not appear to have a direct connection to suicide. The review includes papers that address more general mental health issues, such as depression, that are related to suicide but not specific to suicide. Additional clarity is needed on that point. Second, there is no clear standard for how the efficacy or effectiveness of AI should be judged and the paper tends to repeat the judgements in the papers being reviewed without explaining how those researchers arrived at those judgements. Were AI interventions compared with human therapists, outcome data on actual suicides, symptom reduction? It would be a valuable contribution to describe and assess the ways in which researchers are evaluating AI impacts on suicide-related outcomes, which are notoriously extremely difficult.

# Reviewer report: Review: Are artificial intelligence chatbots safe for suicide risk assessment? A narratively synthesized review of current evidence — R0/PR4

**DOI:** 10.1017/gmh.2026.10260.pr4

The authors have provided a comprehensive review of the existing small body of literature on AI identification of suicidal risk. While the authors describe the limitations of AI to identify suicide risk and suggest they may deployed as complimentary tools for clinicians, they do little to suggest how models may be trained on clinical best practices or salient contextual information. The study by Lee for instance, only used a short free text submission by the patient for AI assessment, and did not include any clinical information, so was obviously only a brief first pass at SI detection. I would have liked to see a discussion on suggested methods and training data sets for AI detection of suicidal ideation and risk.

# Editor report: Recommendation: Are artificial intelligence chatbots safe for suicide risk assessment? A narratively synthesized review of current evidence — R0/PR5

**DOI:** 10.1017/gmh.2026.10260.pr5

**Affiliations:** Department of Primary Care & Mental Health, University of Liverpool, UK, United Kingdom of Great Britain and Northern Ireland

Dear Authors 

We have received reviewer’s comments and we recommend major revision of the manuscript.

Only then can this be considered suitable for publication. 

Regards

Siham

# Editor report: Decision: Are artificial intelligence chatbots safe for suicide risk assessment? A narratively synthesized review of current evidence — R0/PR6

**DOI:** 10.1017/gmh.2026.10260.pr6

**Affiliations:** London School of Hygiene & Tropical Medicine, United Kingdom of Great Britain and Northern Ireland

No accompanying comment.

# Editor report: Recommendation: Are artificial intelligence chatbots safe for suicide risk assessment? A narratively synthesized review of current evidence — R1/PR8

**DOI:** 10.1017/gmh.2026.10260.pr8

**Affiliations:** Department of Primary Care & Mental Health, University of Liverpool, UK, United Kingdom of Great Britain and Northern Ireland

Dear Authors

Thanks for addressing reviewers comments and submitting the revised manuscript. 

We accept it for publication. Congratulations. Our team will be in touch about the next steps

Regards

Siham

# Editor report: Decision: Are artificial intelligence chatbots safe for suicide risk assessment? A narratively synthesized review of current evidence — R1/PR9

**DOI:** 10.1017/gmh.2026.10260.pr9

**Affiliations:** London School of Hygiene & Tropical Medicine, United Kingdom of Great Britain and Northern Ireland

No accompanying comment.
